# Maternal gut microbes shape the early-life assembly of gut microbiota in passerine chicks via nests

**DOI:** 10.1186/s40168-020-00896-9

**Published:** 2020-09-11

**Authors:** Cheng-Yu Chen, Chih-Kuan Chen, Yi-Ying Chen, Andrew Fang, Grace Tzun-Wen Shaw, Chih-Ming Hung, Daryi Wang

**Affiliations:** 1 Biodiversity Research Center, Academia Sinica, Taipei, 115201 Taiwan; 2 Department of Pathology, University of Southern California, Los Angeles, CA 90033 USA; 3 The IEGG and Animal Biotechnology Center, National Chung Hsing University, Taichung, 402204 Taiwan; 4 Department of Molecular Biology and Human Genetics, Tzu Chi University, Hualien, 970301 Taiwan

**Keywords:** Avian microbiota, Host-microbiome interactions, Maternal effects, Gut microbiota assembly, Maternal microbial transmission

## Abstract

**Background:**

Knowledge is growing on how gut microbiota are established, but the effects of maternal symbiotic microbes throughout early microbial successions in birds remain elusive. In this study, we examined the contributions and transmission modes of maternal microbes into the neonatal microbiota of a passerine, the zebra finch (*Taeniopygia guttata*), based on fostering experiments.

**Results:**

Using 16S rRNA amplicon sequencing, we found that zebra finch chicks raised by their biological or foster parents (the society finch *Lonchura striata domestica*) had gut microbial communities converging with those of the parents that reared them. Moreover, source-tracking models revealed high contribution of zebra finches’ oral cavity/crop microbiota to their chicks’ early gut microbiota, which were largely replaced by the parental gut microbiota at later stages. The results suggest that oral feeding only affects the early stage of hatchling gut microbial development.

**Conclusions:**

Our study indicates that passerine chicks mainly acquire symbionts through indirect maternal transmission—passive environmental uptake from nests that were smeared with the intestinal and cloacal microbes of parents that raised them. Gut microbial diversity was low in hand-reared chicks, emphasizing the importance of parental care in shaping the gut microbiota. In addition, several probiotics were found in chicks fostered by society finches, which are excellent foster parents for other finches in bird farms and hosts of brood parasitism by zebra finches in aviaries; this finding implies that avian species that can transfer probiotics to chicks may become selectively preferred hosts of brood parasitism in nature.

Video Abstract

## Background

The gastrointestinal tract is now recognized as being largely sterile in newborn vertebrates [1], and is subsequently colonized by diverse bacterial taxa varying in abundance and functional traits [2, 3]. These microbial communities, termed gut microbiota, may influence a wide range of metabolic, developmental, and physiological processes, affecting host health, fitness, and even behavior [4–6]. The transmission routes of symbiotic microbes—such as neonatal delivery, diet, environment, and parenting behavior—may shape the pool of potential colonists in gut microbiota [7–13]. On the other hand, inherited host-associated factors—such as genotype, sex, and immune status—may function as selective filters in the process of gut microbial community assembly [14–16].

Transmission routes have fundamental effects on microbial symbiont persistence and evolution [17]. Gut microbiota are transferred both from mother to offspring [8] and via other resources through social interactions, shared environment, and diet [11, 18–20]. Even though maternal transmission *sensu stricto* refers to direct transmission before birth, it is increasingly common for studies to also include indirect routes of maternal transmission [21], which now encompass any transfer of maternal symbionts to offspring during or after birth. In viviparous mammals, the initial colonists of the newborn gut come from maternal vaginal, fecal, and breast milk microbes; later on, a great degree of parental care may add diverse parental microbes, such as skin microbes, to newborns during their early stage of gut microbial community development [1, 22–24]. This process is crucial to the recruitment and establishment of neonatal microbiota and aid in defending against pathogens when the immune system is immature [25–28].

In birds, given that embryos develop in eggs—a closed and essentially sterile environment [29]—the chick gut may acquire microbes from eggshells [30, 31], maternal cloacal and fecal microbes smeared in the nest, or parents’ oral microbes through feeding [29, 32]. These transmission routes are especially important in altricial species, including passerines, because their chicks totally depend on parental care for survival [33]. Altricial chicks are typically raised in nests and fed with food that their parents catch or regurgitate. Although studies have suspected that eggshells carry the maternal microbes to chicks, no direct evidence for this has been found [31]. On the other hand, a recent study showed that the microbes of nests resembled those of internal (cloaca) and external (skin and feather) body sites in two lark species (*Lullula arborea* and *Alauda arvensis*) [34]. However, the roles of nests, eggshells, and oral feeding in mediating the early-life assembly of bird gut microbiota are still largely unclear.

A growing number of studies have investigated the transmission routes of early-life gut microbiota in natural populations of birds. In particular, avian brood parasitism, one of the most bizarre breeding strategies, provides a unique natural system for exploring transmission processes. Interspecific brood parasites lay their eggs in the nests of hosts, which incubate the parasitic eggs and raise the chicks. Therefore, parasitic chicks may acquire their gut microbiota from their biological mothers via direct transmission, foster parents via external (indirect) transmission, or both. Studies on parasitic cuckoos (*Clamator glandarius*) and their host magpies (*Pica pica*) suggest that genetic components are important in chick gut microbiota assembly, as magpie and cuckoo nestlings raised in the same nests have different gut microbial communities [35, 36]. In contrast, a study on parasitic cowbirds (*Molothrus ater*) suggested that geographic location and diet might exert strong effects on avian gut microbiota [37]. In addition, cross-fostering experiments in the field suggested that nesting environments may shape the cloacal bacterial assemblages in great tit (*Parus major*) and blue tit (*Cyanistes caeruleus*) nestlings; however, the lack of microbiota data on foster parents and nests prevented them from disentangling the confounding effects of the two components [38, 39]. Overall, current research findings on the transmission routes and establishment of gut microbial communities in hatchlings are still controversial, as are possible changes in these routes over the course of chick development. Fostering experiments with careful designs in well-controlled conditions will be useful for addressing these issues.

The zebra finch (*Taeniopygia guttata*) is a well-established songbird model for animal behavior, neurobiology, and physiology [40, 41]. The society finch (*Lonchura striata domestica*) belongs to the same family of zebra finches, and has been bred over hundreds of years to be foster parents for other finch chicks, including zebra finches. Hence, a zebra finch–society finch fostering experiment in a well-controlled laboratory would be a good system to examine the complicated routes of gut microbiota transmissions. Interestingly, zebra finches were shown to lay their own eggs in society finch nests after their own nest was experimentally destroyed in aviaries; this behavior may provide a window into the evolution of brood parasitism in birds [42].

**Fig. 1:**
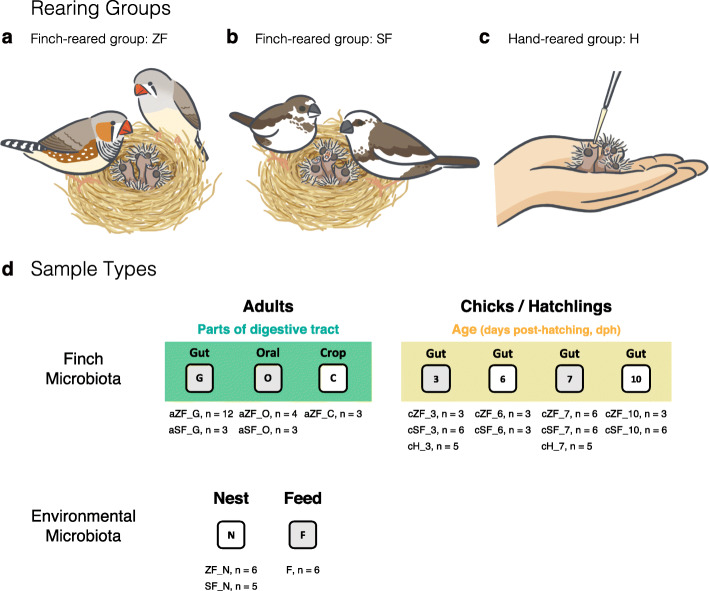
The fostering experiment on the zebra finch–society finch system. We performed a cross-reared experiment using the zebra finch (ZF)–society finch (SF) fostering system and sampled the gut contents of zebra finch hatchlings that were (**a**) raised by their biological brood parents (ZF-reared chicks), (**b**) foster parents (SF-reared chicks), or (**c**) hand-reared (the control group, excluding maternal influence). (**d**) Sampling design of the cross-reared experiment. Illustration by Hsiang-Ching Chen

In this study, we aimed to examine the relative importance of genetic and maternal effects on the newborn gut microbiota. We further examined whether the maternal transmission of the chick gut microbiota mainly occurs through oral feeding (bacteria from parental oral cavities) or fecal contact in nests (bacteria from parental guts) across the different stages of chick development. We applied high-throughput sequencing to 16S rRNA gene amplicons from the gut microbiome of zebra finch hatchlings raised by their biological parents, foster parents (i.e., society finches), and humans (Fig. 1), as well as from the oral cavity, crop, and gut microbiomes of the parents. Our results will also shed a new light on the gut microbiota assembly process during the initial phase of avian brood parasitism evolution.

## Methods

### Finch fostering experiment and microbiota sample collection

Pairs of adult finches used in this study were purchased from a breeder in Tainan, Taiwan (N 23° 14′ 09.7″, E 120° 16′ 53.1″) and kept in a bird room at Biodiversity Research Center of Academia Sinica, Taipei, Taiwan, for at least 1 month before the experiments began. We maintained the bird room at 23–25 °C, ~ 40% humidity, and a day–night cycle of 10 h: 14 h (as described in [41]). We housed each breeding pair in a wire cage, with dimensions of 45.5 × 37.0 × 41.5 cm, and provided them with sterile coconut fibers as nesting materials. The seed component of the feed for all finches including adults and hatchlings comprised a mixture (by volume) of white millet (50%) and canary seed (50%), plus Niger seed (20 g per liter).

To investigate the contributions of maternal microbes to the early development of the gut microbiota in zebra finch hatchlings, we performed a fostering experiment on the zebra finch (ZF)–society finch (SF) system. Freshly laid eggs were collected from six pairs of adult ZF and randomly placed in the nests of SF or an external egg incubator (at 38 °C and 40% relative humidity; Octagon 20 Advance Semi, Brinsea Products Ltd, Standford, UK). After the egg collection, each zebra finch clutch consisted of 2–4 eggs, and the six ZF pairs continued to incubate the remaining eggs and then rear newborn chicks until a given post-hatching stage. To collect finch symbiotic microbiota, we anesthetized finches with isoflurane (Halocarbon Products Corporation, Peachtree Corners, GA, USA) prior to decapitation. After sacrificing the birds, we sampled the gut (including intestine and cloaca) contents of zebra finch hatchlings raised by their biological parents (ZF-reared chicks, cZF: *n* = 15) or foster parents (SF-reared chicks, cSF: *n* = 21) at four different developmental stages: 3, 6, 7, and 10 days post-hatching (dph) (see details in Fig. 1). To create a control group that excluded maternal transmission via the nest environment and regurgitated food (but not eggshells), the incubator-hatching chicks (cH: *n* = 10) were hand reared with homogenized feed (FastPrep-24™ Homogenizer, MP Biomedicals) using a micropipette with sterile filtered pipette tips every 2–4 h, sampled at 3 and 7 dph. We also sampled the oral swab (FLOQSwabs, Copan, Italy), crop, and gut contents of adult zebra finches (aZF: *n* = 12) and society finches (aSF: *n* = 3), as well as their nest materials (N: *n* = 11) from the surface of nest bottoms, which the finches came in contact with most often, and feed samples (F: *n* = 6). Comparing gut community dynamics in finch-reared and control hatchlings allowed us to examine the influences of parental care and nesting environment on microbiota acquisition at that stage of hatchling development. All samples were stored at −20 °C until DNA extraction.

### DNA extraction, amplification, and metagenomic sequencing

Samples harvested after the birds were sacrificed were homogenized in 1.4 mL of lysis buffer (Buffer ASL, QIAamp DNA Stool Mini Kit) using FastPrep-24 Homogenizer (MP Biomedicals) in Precellys Lysing Kit CK14 tubes (Bertin Technologies, Montigny le Bretonneux, France). The bacterial DNA from homogenized finch (gut, crop, and oral) and environmental (nest materials and feed) samples were extracted using the QIAamp DNA Stool Mini Kit (QIAGEN, GmbH, Hilden, Germany) and quantified using a Qubit 2.0 Fluorometer (Invitrogen, Life Technologies, CA, USA).

The first two hypervariable regions (V1-V2) of the small subunit ribosomal RNA (16S rRNA) gene were amplified using universal eubacterial primers. The forward primer 27F (5′-AGAGTTTGATCMTGGCTCAG-3′) and reverse primer 355R (5′-GCTGCCTCCCGTAGGAGT-3′) [43, 44] were fused with Illumina overhang adapters and specific 10-nt barcodes to allow multiple samples to be analyzed in parallel on a single picotiter plate. The pooled DNA was amplified with PCR (Taq DNA Polymerase 2x Master Mix RED, Ampliqon, Odense M, Denmark) under the following running conditions: initial denaturation for 3 min at 95 °C; 30 cycles of 30 s at 95 °C, 30 s at 55 °C, and 45 s at 72 °C; and a final elongation step for 10 min at 72 °C, modified from the Illumina standard protocol for 16S metagenomic sequencing library preparation [45]. All PCR products were confirmed using 2% agarose gel electrophoresis and subsequently isolated from the gel and purified by NucleoSpin Gel and PCR Clean-up (Macherey Nagel, Düren, Germany). DNA concentrations of the clean PCR products were determined using a Quant-iT dsDNA HS assay kit and Qubit fluorometer (Invitrogen, Carlsbad, CA, USA). The purified amplicons were further processed according to the Illumina standard protocol, and paired-end 2 × 300 bp sequencing was conducted on the MiSeq platform (Illumina, San Diego, CA, USA) with the reagent kit v3 at the NGS High Throughput Genomics Core Facility at Academia Sinica. All datasets were deposited into the Sequence Read Archive (SRA) database at NCBI under BioProject ID PRJNA609776. A full list of sample identifiers is provided in Supplementary Table S1 (Additional file 2).

### Sequence data processing

The raw Illumina amplicon reads were demultiplexed and merged with the FLASH software [46], then quality filtered and analyzed using mothur v1.35.1 [47]. The criteria for filtering were minimum read length of 200 bp, maximum read length of 600 bp, minimum sequence quality score of 30, maximum number of errors in the barcode of 1 bp, and maximum number of errors in the primer of 2 bp. Barcode and primer sequences were removed from the 5′ and 3′ ends, and chimeras were searched for and removed using the *uchime_ref* command in USEARCH [48]. After filtering and trimming, reads with an average length of 307 bp among all samples were used for downstream analyses.

A UPARSE pipeline (*usearch_global*) [49] was used to cluster preprocessed reads into operational taxonomic units (OTUs) at 97% similarity. The OTUs were further assigned into a taxonomic hierarchy based on the reference sequences from the Greengenes database (version 13.8.99) using mothur (*classify.seqs*) [50]. Sequences that were classified as chloroplast, mitochondria, eukaryota, or unknown were removed from the dataset (*remove.lineage*), as they likely represented ingested plant materials. Samples were rarefied to the minimum of 9557 sequences before being used for diversity analysis to avoid biases caused by uneven sequencing efforts.

### Statistical analysis

We calculated the observed (OTU) richness and Shannon diversity (i.e., alpha diversity) index from rarefied data using mothur and applied analysis of variance (ANOVA) with Tukey-Kramer post hoc tests to analyze differences in microbiota diversity between rearing groups (i.e., hand-, SF-, and ZF-reared groups, including both adults and hatchlings) and sample types (i.e., guts, crops, oral cavities, nests, and feed). The adjusted *P* values for pairwise Tukey-Kramer contrasts were reported from the default single-step method in the R package *multcomp* [51].

To assess the compositions of bacterial communities among groups or sample types (i.e., beta diversity), we used Bray-Curtis dissimilarities and weighted UniFrac [52] to evaluate phylogenetic similarities. The non-metric multidimensional scaling (NMDS) ordination approach [53] based on Bray-Curtis dissimilarities from the OTU-level table was conducted using the R package *vegan* [54] to perform a parallel comparison. Apparent changes in community structure were tested using analysis of molecular variance (AMOVA) [55] in mothur.

The average relative abundance of the most prevalent bacterial families (abundance > 0.5%) was estimated for each sample type and plotted for samples from the three rearing groups and feeds. To further measure the specificity of a bacterial taxon (at the family level) to a given sample type, we determined its indicator value (IndVal) index, which considers the relative abundance of a taxon in a given community and its relative frequency of occurrence across all communities. The IndVal index quantified the specificity of taxa from those found in only a single community to those found across all communities. Bubble plots of relative taxonomic abundances and IndVal were generated using the R package *ggplot* to assess the impacts of microbiota on adult digestive systems, feed, and nest materials on those in chick guts.

To identify significantly different taxa among hatchlings from hand-, SF-, and ZF-reared groups, the linear discriminant analysis (LDA) effect size (LEfSe) was performed using the online Huttenhower Galaxy server (http://huttenhower.sph.harvard.edu/galaxy/). In this analysis, LEfSe determined the active bacterial taxa (from the phylum to genus levels) in the finch gut microbiome based on changes in OTU abundance to explain differences among development stages and rearing groups. We then used SourceTracker2 [56], a Bayesian community-level microbial source-tracking tool, to estimate the proportion of sequences in the hatchling gut microbiota that originated from their biological parent, foster parent, or environmental communities. SourceTracker2 was run with default parameters using non-rarefied data; each hatchling gut microbial community was designated as a sink, and all other finches and environmental sample types were designated as sources.

## Results

### Richness and diversity of finch and environmental microbiota

Our 16S rRNA sequencing effort produced 6,265,933 quality filtered reads clustered into 2,553 OTUs. Sequence coverage ranged from 9557 to 149,280 reads per sample. The coverage ranges of different sample types were narrower (feed: 9557–39,711, nest: 48,572–130,324, oral: 77,726–131,807, crop: 50,730–122,496, and gut: 10,800–149,280; see rarefaction curves in Additional file 1: Figure S1 and detailed information in Additional file 2: Table S1) than that of the total range.

**Fig. 2:**
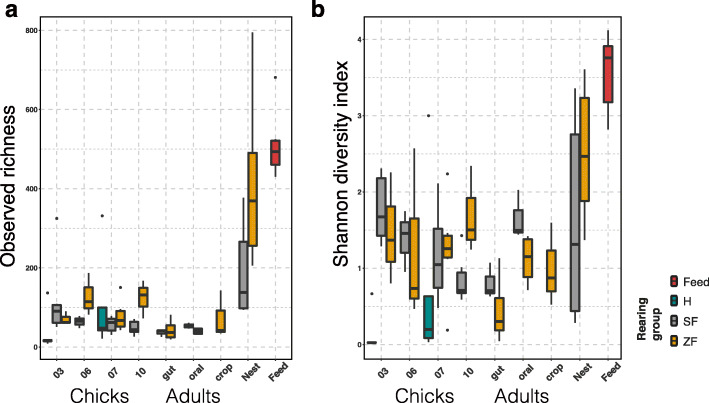
Alpha diversity across sample types of hand- (H), society finch- (SF), and zebra finch (ZF)-reared groups. (**a**) The observed richness and (**b**) Shannon diversity index of microbial communities in samples from the hatchling gut (at 3, 6, 7, 10 days post-hatching, dph), adult digestive tract (gut, oral, crop), and environment (nest materials and feed). The hand-reared group included only hatchling gut samples at 3 and 7 dph

We found that the finch and environmental microbiota varied across different sample types. The environmental samples (nest and feed) harbored higher OTU richness and more diverse microbiota than finch samples from the different parts of adult digestive tracts (oral cavities, crops, and guts) and from the guts of hatchlings at different days post-hatching in both finch-reared groups (Fig. 2). In finch gut microbiota, observed OTU richness was not significantly different between the two finch-reared groups, including parents and hatchings (SF-ZF: ANOVA, *F*_1,49_ = 0.048, *P* = 0.83), or among hatchlings raised by human and finch parents (cH-cF: *F*_1,44_ = 0.153, *P* = 0.698) (Fig. 2a). Nevertheless, the finch-reared hatchlings had significantly higher alpha diversity (Shannon index) levels in their gut microbiota than those of hand-raised ones (cH-cF: *F*_1,44_ = 11.920, *P* = 0.001).

Age had a major impact on finch gut microbial diversity, as we observed modest reductions in OTU richness (chicks-adults: *F*_1,59_ = 5.224, *P* = 0.026) and strong decreases in Shannon index (chicks-adults: *F*_1,59_ = 9.832, *P* = 0.002) from hatchings to adults. Among finch-reared hatchlings, the Shannon diversity of chicks raised by society finches continued to decrease over the first 10 days after hatching, whereas those of ZF-reared chicks did not show such an age-associated decline (Fig. 2b).

### Early-life finch gut microbial community structure

**Fig. 3:**
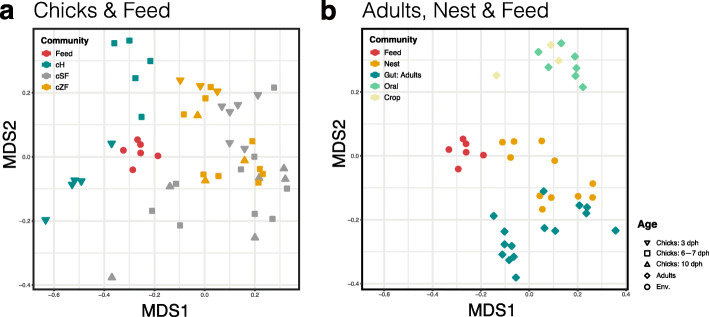
Microbial community structures across finch and environmental samples. Non-metric multidimensional scaling (NMDS) ordination on Bray-Curtis distances among sample types was plotted based on OTU abundances in (**a**) chick, (**b**) adult, and environmental (Env.: nest and feed) samples. cH, cSF, and cZF: ZF chick gut microbiota from hand-, society finch-, and zebra finch-reared groups

We next sought to examine how the hatchling gut microbial communities varied across different rearing groups at 3, 6, 7, and 10 days post-hatching (dph) (Fig. 3 and Additional file 1: Figure S2). The SF- and ZF-reared chicks did not show distinct gut microbial community structures at any age (Fig. 3a). On the other hand, the gut microbiota in the hand-raised chicks were somewhat clustered and separated from those of finch-reared chicks (cH-cF: AMOVA, *Fs*_1,44_ = 7.019, *P* < 0.001), while the feed microbiota was the closest bacterial community to the former (Fig. 3a). Pairwise Bray-Curtis dissimilarity comparisons between communities (beta diversity) further corroborated these observations. The beta diversities of microbiota between finch-reared chicks (cSF and cZF) and samples obtained from their parents and nest environment were significantly lower than those between hand-reared chicks (cH) and other samples (Additional file 1: Figure S3, dashed line versus solid line). In the finch-reared hatchlings, adult oral and crop communities were significantly different from 10-dph chick gut communities (aFO-cF10: *Fs*_1,14_ = 4.422, *P* < 0.001; aFC-cF10: *Fs*_1,10_ = 2.835, *P* = 0.005), but were less different from the 3-dph ones (aFO-cF3: *Fs*_1,14_ = 2.903, *P* = 0.003; aFC-cF3: *Fs*_1,10_ = 1.995, *P* = 0.06) (Fig. 3); in contrast, adult gut communities were more different from 3-dph chick gut communities (aFG-cF3: *Fs*_1,22_ = 9.088, *P* < 0.001) than from 10-dph ones (aFG-cF10: *Fs*_1,22_ = 6.445, *P* = 0.004) (Fig. 3a, b).

**Fig. 4:**
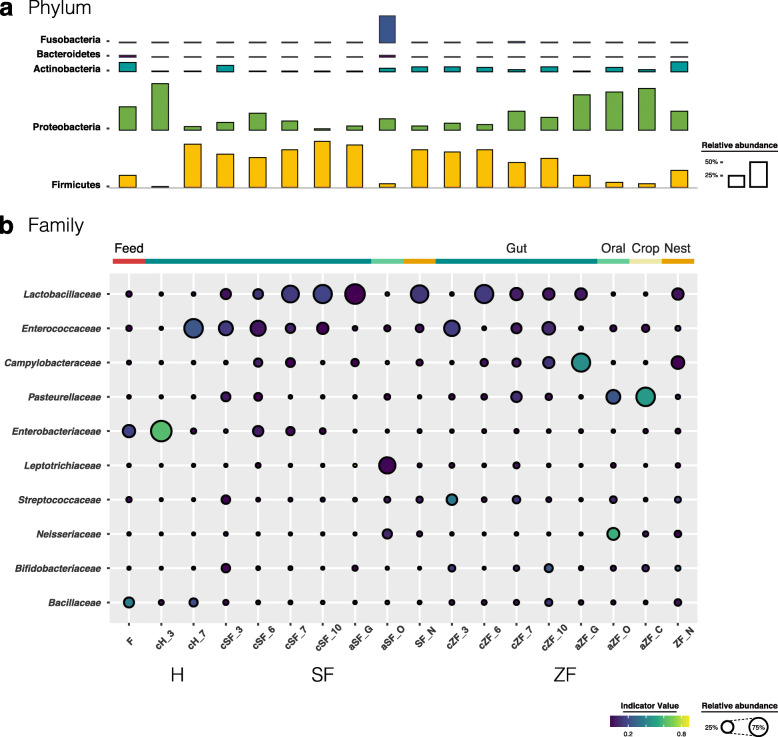
Microbial compositions of the finch and environmental microbiota. The average relative abundances of the most prevalent bacterial (**a**) phyla (bar length) and (**b**) families (circle size) in each sample type (indicated as above bubble plot) plotted for samples from the three rearing groups and feed. The indicator value index (shading of circle color) represents the strength of association between a taxon and a given sample type, with larger values indicating greater specificity

Of the 30 total identified bacterial phyla, six dominated the finch gut microbiota (average cumulative abundance = 99.5%), including the phyla shown to be prevalent in avian gut communities—Firmicutes, Proteobacteria, Actinobacteria, and Bacteroidetes—in other studies [57] (Fig. 4a). Firmicutes comprised the most dominant phylum among the hatchling gut microbiota, except in the 3-dph hand-reared chicks. However, within these Firmicutes-dominated communities, the three rearing groups possessed different distributions at the family level (Fig. 4b and Additional file 1: Figure S4). *Lactobacillaceae* and *Enterococcaceae* were prevalent in the finch-reared hatchlings. SF-reared chicks harbored on average the largest fraction of *Lactobacillaceae*, the proportion of which increased over the growth of hatchlings (average abundance from 16.2% at 3 dph to 73.4% at 10 dph), opposite to that of *Enterococcaceae* (average abundance from 38.2% at 3 dph to 22.2% at 10 dph); ZF-reared chicks showed a relatively stable pattern of age variation during growth. Noteworthy, the predominant members of the gut microbiota in adults—*Lactobacillaceae* for the society finch (average abundance = 87.3%) and *Campylobacteraceae* for the zebra finch (average abundance = 73.0%)—were also prevalent in the hatchlings they raised and their nest environments. In contrast, the hand-raised chicks had few signature taxa except *Enterobacteriaceae* and *Enterococcaceae*, which were predominant at 3 and 7 dph, respectively (average abundance = 97.3% and 76.7%, respectively), consistent with the lowest bacterial diversities among all experiment groups (Fig. 2).

To further measure the specificity of a taxon to a given sample type, we determined its indicator value (IndVal) index. For instance, in the hand-reared group, *Enterobacteriaceae* was specific to the chicks at 3 dph (IndVal = 0.616; Fig. 4b), whereas *Bacillaceae* had a relatively higher specific index in the 7-dph chicks (IndVal = 0.205) and feed microbial communities (IndVal = 0.375). In addition, the oral cavity and crop of zebra finch adults were characterized by *Pasteurellaceae* (oral: 36.9%, IndVal = 0.230; crop: 76.6%, IndVal = 0.477), whereas their gut was characterized by *Campylobacteraceae* (IndVal = 0.436); the above two bacterial phyla also occurred in the gut microbiota of the ZF-reared chicks.

### Influence of the maternal microbiota on the early stages of finch gut microbial successions

**Fig. 5:**
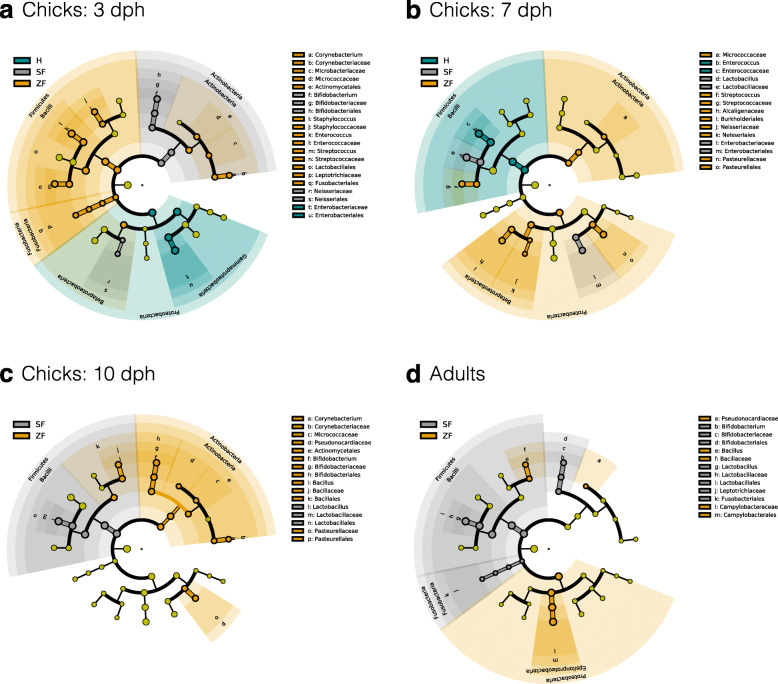
The active bacteria in the finch gut microbiota, determined by LEfSe among three rearing groups. The gut microbiota of (**a**) 3-, (**b**) 7-, (**c**) 10-dph chicks, and (**d**) adults from hand (H)- (green), society finch (SF)- (gray), and zebra finch (ZF)-reared group (gamboge yellow) were compared. Colors indicate taxa or branches of the tree, which more significantly represent certain groups than others, except that gray yellow circles indicate taxa with non-significant differences

To determine the association between early gut microbial successions and parental care, linear discriminant analysis effect size (LEfSe) analysis [58] was performed to identify representative taxa in given communities. An appreciable resemblance was detected between the gut microbiota of the hatchlings and their feeding parents (Fig. 5 and Additional file 1: Figure S5). Consistent with the microbial composition results (Fig. 4b), the LEfSe results showed that the adult gut microbiota of society finches enriched in *Lactobacillaceae*, *Bifidobacteriaceae,* and *Leptotrichiaceae,* while that of zebra finches enriched in *Bacillaceae* and *Campylobacteraceae* (at the family level; Fig. 5d). In the SF-reared chicks, *Bifidobacteriaceae* and *Neisseriaceae* were dominant at 3 dph (Fig. 5a). By 7–10 dph, had *Lactobacillaceae* increased appreciably and become the dominant bacterial family (Fig. 5b, c). In contrast, *Streptococcaceae*, *Neisseriaceae*, and *Pasteurellaceae* were relatively abundant in the 7-dph ZF-reared chicks, and then *Bacillaceae* and *Pasteurellaceae* became dominant at 10 dph (Fig. 5c). In the hand-raised group that was designed to eliminate (external) maternal transmission, only *Enterobacteriaceae* and *Enterococcaceae* were predominant in chicks at 3 and 7 dph, respectively (Fig. 5a, b). Thus, the LEfSe results of taxa at the family level further demonstrated that maternal gut microbes were the substantial contributor to early-life successions in the finch gut community.

### Predictive source tracking of hatchling gut communities

**Fig. 6:**
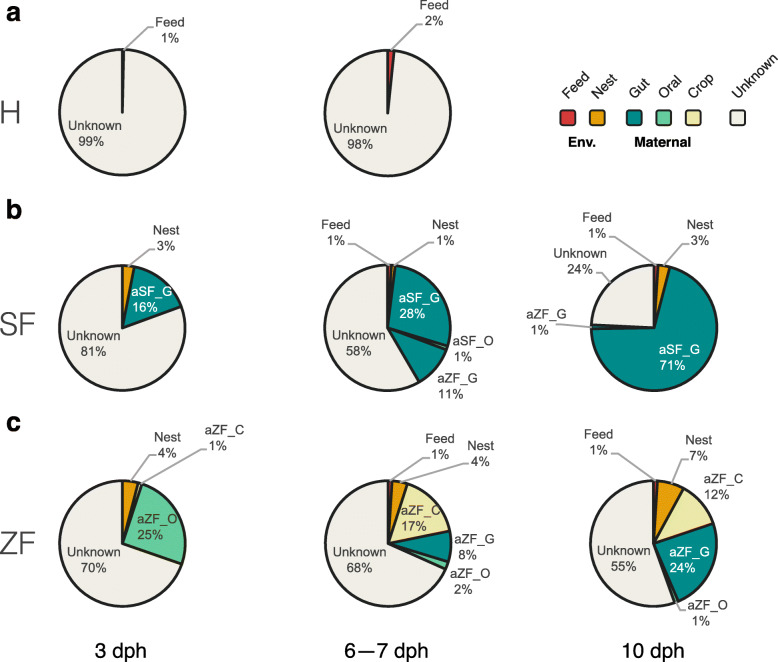
Community-level source-tracking models for the maternal and environmental sources of early gut community assembly. The predicted proportions of sequences in the hatchling gut microbiota of (**a**) hand (H)-, (**b**) society finch (SF)-, and (**c**) zebra finch (ZF)-reared chicks at different days after hatching (3, 6–7, and 10 dph) that originated from their biological parents, foster parents, or environmental communities

We finally used Bayesian community-level source tracking [56] to investigate how maternal or environmental sources contributed to the gut community assembly of hatchlings. The SourceTracker model also revealed the major role of maternal gut microbes in shaping finch-reared chick gut microbial communities, especially at 10 dph (Fig. 6b, c). On average, 71% of the SF-reared chick gut microbiota were from maternal gut sources, 3% from nesting environmental sources, less than 1% from feed, and 24% from unknown sources; on average, 24% of the ZF-reared chick gut microbes were from maternal gut sources, 12% from maternal crop sources, 7% from the nest, and 55% from unknown sources. The results were consistent with the patterns derived from the taxonomy and linear analyses (Figs. 3, 4, 5, and Additional file 1: Figure S2). Despite a noticeable level of compositional mismatch between ZF-reared chick gut microbiota and considered source communities, SourceTracker identified maternal oral and crop communities as dominant sources of chick gut communities from 3 to 7 dph. In contrast, maternal oral and crop communities contributed little to the gut communities of SF-reared chicks at any stage. Using SourceTracker to estimate the origin of hand-reared chick gut microbiota, we found to our surprise little evidence that feed microbes colonized hatchling guts, which had microbiota with largely unknown sources (Fig. 6a; see the “Discussion” section for details).

## Discussion

Although still scarce, several studies in recent years have investigated the early establishment of the gut microbiota in birds such as chickens [32], ostriches [59], and shorebirds [29]. Avian brood parasitism and fostering experiments in the field [36, 37, 39] have been used to evaluate the maternal and environmental effects on the gut microbial assembly of newborns. However, previous studies based on natural populations often show conflicting results, probably due to the confounding effects of parental (feeding behaviors) and environmental (available food or habitats) microbial transmission. Here, we studied the zebra finch–society finch fostering system in a well-controlled environment as an alternative strategy to further determine microbial transmission routes across several stages of hatchling ontogeny. Our results demonstrate that the gut microbial community of newborn zebra finches resembles that of the adult finches that reared them. That is, chick guts obtain and accumulate parental gut microbes after hatching, and genetics do not play an important role in this process. Our study also provides the first evidence that avian hatchling gut microbiota are mainly shaped by the transmission of parental gut microbiota via nests, especially at later stages of chick development.

### Nesting environment as a crucial route for maternal transmission to shape the hatchling gut microbial community

Chicks generally harbor more diverse gut microbiota than their parents, and the differences decrease as they grow. In the SF-reared group, the alpha diversity and richness of hatchling gut microbiota are the highest in neonatals (3-dph chicks), and subsequently decrease during maturation. Our results are in contrast to the pattern of mammal gut microbial establishment; mammal gut microbial diversity increases progressively beginning with the initial colonization during delivery because parental care supplements the young with parental microbes [1, 60]. However, several studies on wild bird species [29, 39, 61, 62] congruent with our results suggest that newborns already harbor diverse gut microbiota owing to the rapid colonization of gut microbes shortly after hatching. The high microbial diversity of hatchling gut communities might come from not only the biological mother (via the eggshell), but also feeding parents through direct contact during food provisioning (via maternal oral cavities or crops) or passive exposure to the nesting environment (via maternal feces). We further discuss the relative importance of these three transmission routes using our analytical results.

We found little evidence that the biological mother transmits her gut microbiota via eggshells. A previous study found that eggshells have bacterial communities strongly resembling those of the maternal skin, feather, and nest material, and therefore may act as a transgenerational carrier of maternal microbiota to the hatching offspring [31]. However, the hatchlings in our control group, for which possible maternal transmission is confined to eggshells, did not harbor gut microbiota resembling those of their biological mothers. Instead, the gut microbiota of 3-dph hand-reared hatchlings had low diversity and richness, and only shared a few bacteria with its feed. The results suggest a trivial role of eggshells in transferring maternal symbiotic microbes for the initial establishment of the avian gut microbiota. The low gut microbial diversity of hand-reared chicks also emphasizes the importance of parental feeding and/or nesting environment in gut microbiota establishment.

Maternal transmission via regurgitated food is relatively important at the early stage of zebra finch development, and its impact diminishes as chicks grow. This argument is supported by the results that zebra finch parents’ oral cavity/crop microbiota strongly contributed to chicks’ early gut microbiota, which were largely replaced by parental gut microbiota at later development stages (Fig. 6). However, the gut microbiota of fostered chicks was mainly contributed by the gut microbiota of society finches throughout their development. Thus, maternal transmission via regurgitated food is only observed in chicks reared by their biological parents, not in those reared by foster parents. The results imply that inter-species differences in immune systems or physiological factors may determine the role of oral feeding on early gut microbiota establishment in chicks.

Most importantly, our data suggest that the gut microbiota of hatchlings raised by finches is assembled mainly through maternal transmission via the nesting environment. We found that the nest microbiota significantly resembled the microbial composition of adult guts, and as hatchlings stayed in nests longer their gut microbiota converged with that of the parents that reared them. Nests often accumulate parental feces, which likely contain bacteria from their guts. Hence, our results suggest that the nesting environment may function as a transgenerational carrier of maternal gut microbes—a route for maternal transmission that has not been directly tested before [38, 39].

### Maternal effect on gut microbiota assembly during hatchling development

Alongside the decrease in microbial diversity over hatchling development, the contributions of the maternal gut community substantially increased in finch-reared groups. Thus, maternal gut symbionts are the major contributors to the establishment of avian gut microbiota. The predicted active and dominant bacterial families in the adult gut—*Campylobacteraceae* in the zebra finch and *Lactobacillaceae* in the society finch—also became prevalent in the hatchlings they raised at 10 dph. In the ZF-reared group, the major shift in the hatchling gut microbial composition was from *Enterococcaceae* at 3 dph to *Campylobacteraceae* at 10 dph. *Campylobacter* and related genera in the family *Campylobacteraceae* are oral and intestinal commensals of vertebrates [63], and may be one of the important sources of mortality and morbidity in wild and domestic animal populations [64]. However, *Campylobacter* spp. are generally accepted to be nonpathogenic in avian hosts [65]; they were consistently isolated from the guts of zebra finches that were outwardly healthy [66]. In the SF-reared group, the major bacterial family changed from *Bifidobacteriaceae* at 3 dph to *Lactobacillaceae* at 10 dph. Many gut symbiotic microbes in these two families are considered as probiotics that play an important role in nutrition, growth, and protection from infection [4, 67, 68]. Both microbial families are the LEfSe-predicted active bacteria in adult society finches. This finding supports a common opinion in pet bird farms that the society finches are excellent foster parents for other finches, probably because they can maintain a healthy gut microbiota in hatchlings during early-life development.

In contrast, several candidate probiotics, such as *Lactobacillaceae* and *Bifidobacteriaceae*, were not well established in the gut communities of the hand-reared hatchlings. In fact, the gut microbiota of these hatchlings had a low diversity and richness, and shared the predominant bacterial family *Enterobacteriaceae* with their feed only at 3 dph. *Enterobacteriaceae* may reflect the influence of the Proteobacteria-rich microbiota, which is associated with xylanase for cellulolytic pre-digestion of feed in birds [57]. The hand-raised hatchlings at 7 dph harbored a gut bacterial population dominated only by *Enterococcaceae*, which is one of the prevalent avian intestinal microbes [57, 65, 69, 70] and was also observed in the hatchling gut communities from the finch-reared groups. In the hand-reared group, the feed microbial community seemed to be the major and perhaps only contributor according to the taxonomic composition, although our source tracking results failed to provide statistical support due to a low number of reads in this group. It is possible that feed microbes are important colonists for the hatchlings of all groups, but are disproportionately excluded by the host immune system or gut environment. The reason for the large unknown source in the tracking results is that this analysis does not consider all source-sink scenarios, nor does our study include other potential environmental sources (e.g., the incubator box for the hand-reared group). Nevertheless, our results highlight the importance of parental care in shaping the gut microbiota in birds. Such maternal effects could be critical to the health of chicks.

## Conclusion

The gut microbiota is shaped by multiple factors, and the relative contributions of host genetics and environments remain uncertain. In recent years, the environment has come to be considered far more important than host genetic relationships in determining host gut microbiota [11]. Among the environmental factors, diet has emerged as a pivotal determinant of gut microbiota community structure and function [71, 72]. On the other hand, growing evidence points out that avian gut microbiota are likely determined by habitat locations or the nests in which chicks are reared [31, 34, 37, 39, 73, 74]. In natural populations, the effects of habitat locations, nests, and diet on shaping the microbiota of young chicks are inseparable. Thus, in this study, we controlled the genetics and diet in simplified environmental conditions and used fostering experiments to estimate the maternal effects on the hatchling gut microbiota from 3 to 10 dph. Our results suggest little impact of genetics and diet on the hatchling gut microbiota. We found that young hatchlings initially had gut microbiota distinct from those of adults, and undergo a transition period until reaching a stable, mature adult-like microbial community. Among all the maternal symbionts we collected (from oral cavities, crops, and guts), maternal gut microbes contributed the most to the microbial community of hatchlings, especially toward their late development stages. Noteworthy, our study identified nests, which are often smeared by parental cloacal and fecal bacteria, as a crucial route for maternal transmission on early gut microbial assembly, supporting a notion of “Like mother like nest” [75]. Furthermore, many candidate probiotics were found in finch-reared hatchlings, especially those fostered by society finches, illuminating the positive effects of parental care on gut microbiota assembly during hatchling development. It also implies that wild avian species that can transfer probiotics to chicks may become selectively preferred hosts for parasitic birds like cuckoos and cowbirds. This hypothetical relationship warrants further study.

## Supplementary information

**Additional file 1: Figure S1.** Rarefaction curves across sample types plotted based on OTUs abundance using the R package *iNEXT* [76] and *ggplot2* [77]. **Figure S2.** NMDS ordination on Bray-Curtis distances plotted based on OTUs abundance of all finch and environmental microbiota from three rearing groups. **Figure S3.** Cumulative distribution of Bray-Curtis dissimilarity calculated pairwise between hatchling gut samples from hand- (solid lines) or finch-reared groups (dashed lines) and other samples. **Figure S4.** Bubble plot of average relative abundances and IndVal showing the most prevalent bacterial families in each sample type from three rearing groups. **Figure S5.** Plot LEfSe Results of a) 3-, b) 7-, c) 10-dph hatchling and d) adult gut microbiota from three rearing groups.

**Additional file 2: Table S1.** Metadata for each library in this study.

## Data Availability

The datasets generated and analyzed in the current study are available in the Sequence Read Archive (SRA) database at NCBI under BioProject ID PRJNA609776.

## Abbreviations

AMOVA: Analysis of molecular variance
ANOVA: Analysis of variance
aZF and aSF: Adult zebra finch and society finch
cZF, cSF, and cH: Chick (hatchling) raised by zebra finch, society finch, and human
LEfSe: Linear discriminant analysis effect size
NMDS: Non-metric multidimensional scaling
OTU: Operational taxonomic unit
SF: Society finch
ZF: Zebra finch
