# Supplementary material for: Maternal gut microbes shape the early-life assembly of gut microbiota in passerine chicks via nests
Source: Microbiome. 2020 Sep 11;8:129. doi: 10.1186/s40168-020-00896-9 (PMC7488855; doi:10.1186/s40168-020-00896-9)
Supplement: Supplementary file 2 — Additional file 1: Figure S1. Rarefaction curves across sample types plotted based on OTUs abundance using the R package iNEXT [76] and ggplot2 [77]. Figure S2. NMDS ordination on Bray-Curtis distances plotted based on OTUs abundance of all finch and environmental microbiota from three rearing groups. Figure S3. Cumulative distribution of Bray-Curtis dissimilarity calculated pairwise between hatchling gut samples from hand- (solid lines) or finch-reared groups (dashed lines) and other samples. Figure S4. Bubble plot of average relative abundances and IndVal showing the most prevalent bacterial families in each sample type from three rearing groups. Figure S5. Plot LEfSe Results of a) 3-, b) 7-, c) 10-dph hatchling and d) adult gut microbiota from three rearing groups. [file 40168_2020_896_MOESM1_ESM.docx]

**Additional file 1**

**Supplementary materials**

**Figure S1.** Rarefaction curves across sample types plotted based on OTUs abundance using the R package *iNEXT* and *ggplot2*.

**Figure S2.** NMDS ordination on Bray-Curtis distances plotted based on OTUs abundance of all finch and environmental microbiota from three rearing groups.

**Figure S3.** Cumulative distribution of Bray-Curtis dissimilarity calculated pairwise between hatchling gut samples from hand- (solid lines) or finch-reared groups (dashed lines) and other samples.

**Figure S4.** Bubble plot of average relative abundances and IndVal showing the most prevalent bacterial families in each sample type from three rearing groups.

**Figure S5.** Plot LEfSe Results of **a**) 3-, **b**) 7-, **c**)10-dph hatchling and **d**) adult gut microbiota from three rearing groups.


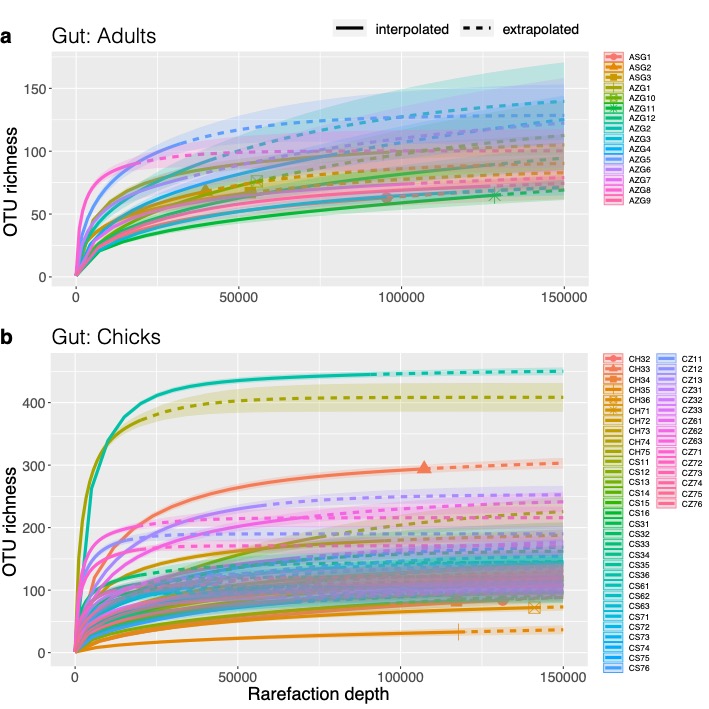


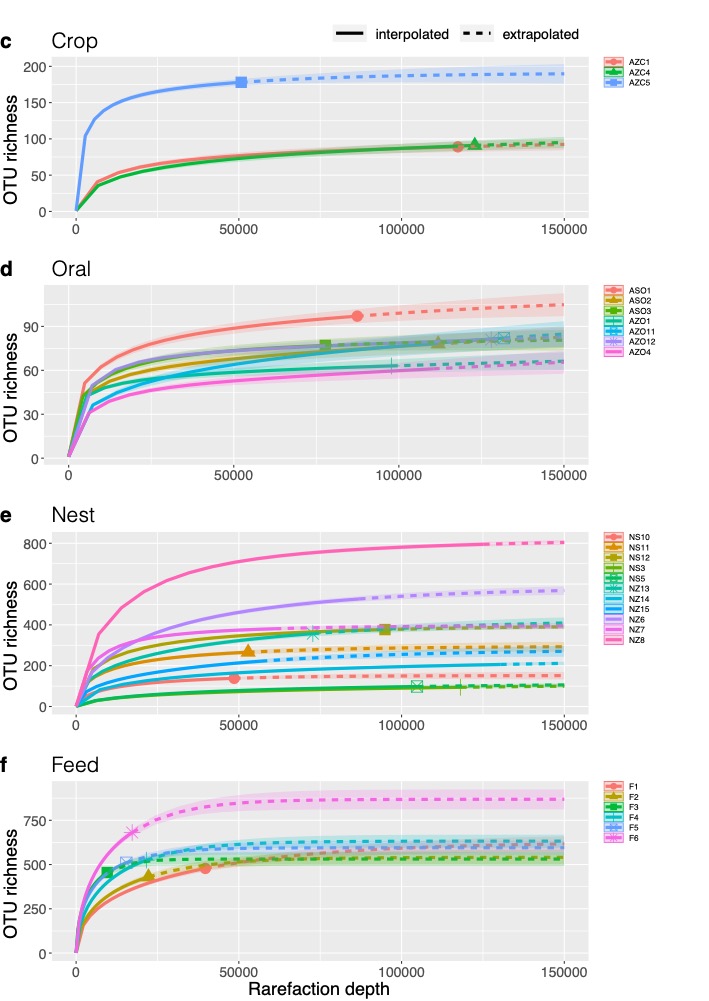


**Figure S1.** Rarefaction curves across sample types plotted based on OTUs abundance using the R package *iNEXT* and *ggplot2*.

**Figure S2.** NMDS ordination on Bray-Curtis distances plotted based on OTUs abundance of all finch and environmental microbiota from three rearing groups. aSF_G, aSF_O, aZF_C, aZF_G, and aZF_O: gut/oral/crop microbiota of adult SF and ZF; cH, cSF, and cZF: ZF chick gut microbiota from hand-, SF-, and ZF-reared groups; SF_N and ZF_N: nest microbiota from SF- and ZF-reared groups; F: feed microbiota; Env.: environmental samples.

**Figure S3.** Cumulative distribution of Bray-Curtis dissimilarity calculated pairwise between hatchling gut samples from hand- (solid lines) or finch-reared groups (dashed lines) and other samples. cH, cSF, and cZF: ZF chick gut microbiota from hand-, society finch-, and zebra finch-reared groups.

**Figure S4.** Bubble plot of average relative abundances and IndVal showing the most prevalent bacterial families in each sample type from three rearing groups.

**a**

**
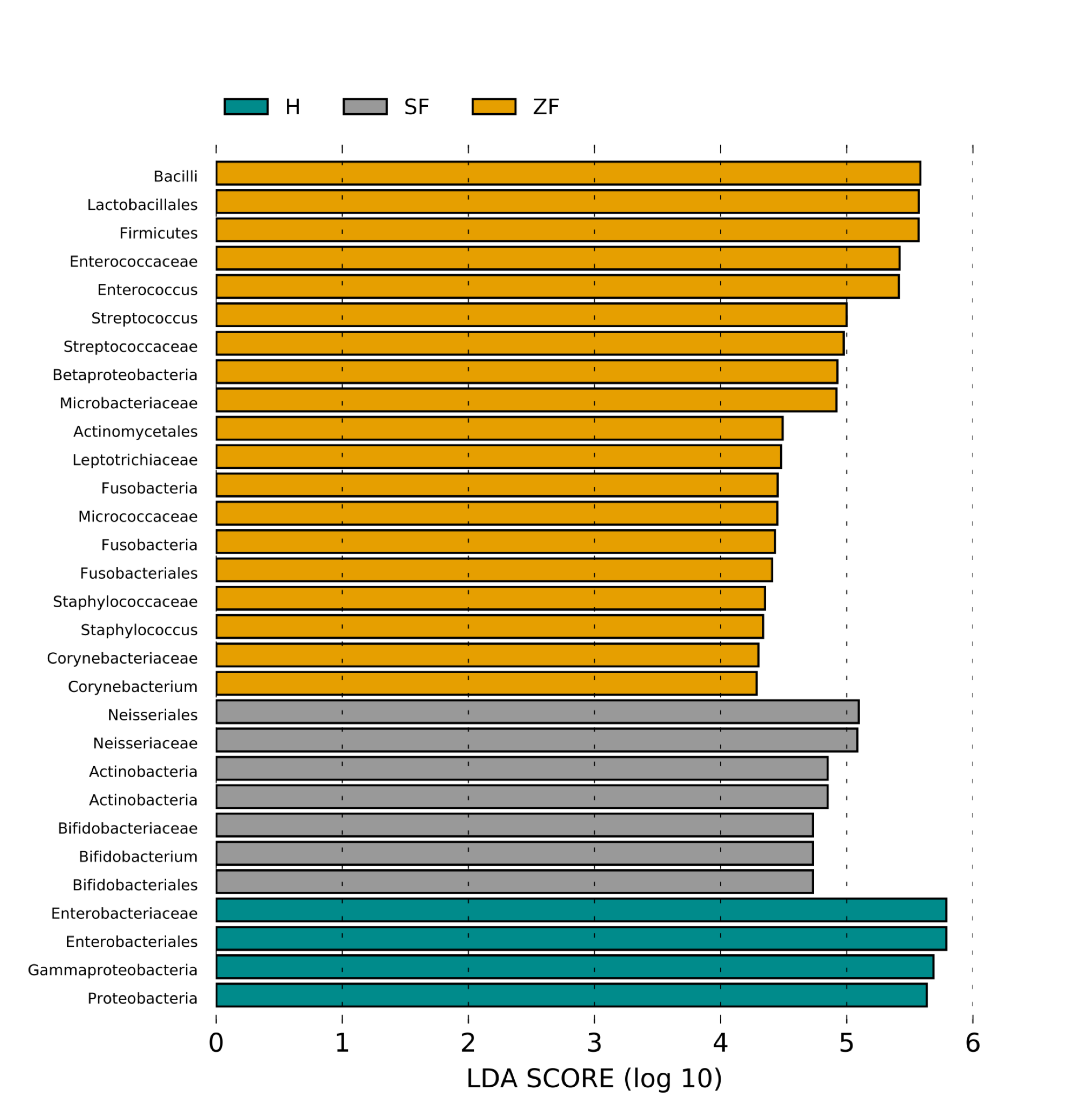
**

**b**

**c**

**d**

**Figure S5.** Plot LEfSe Results of **a**) 3-, **b**) 7-, **c**)10-dph hatchling and **d**) adult gut microbiota from three rearing groups.
